# Distribution of ABO and Rh Blood Groups in Patients With Endometriosis at King Abdulaziz University Hospital: A Case-Control Study

**DOI:** 10.7759/cureus.51268

**Published:** 2023-12-29

**Authors:** Maisam H Alhammadi, Afnan A Alsaif, Dalal A AlGhamdi, Samera Albasri

**Affiliations:** 1 Faculty of Medicine, King Abdulaziz University, Jeddah, SAU; 2 Obstetrics and Gynecology, King Abdulaziz University Hospital, Jeddah, SAU

**Keywords:** late pregnancy, duration of menses, heavy menstrual bleeding, oral contraceptives, endometriosis, rh blood type, abo blood group, infertility, abortion, dysmenorrhea

## Abstract

Background

There is no sound evidence for the association of blood groups with the risk of endometriosis, and no studies from Saudi Arabia have examined this association. Therefore, the primary aim was to determine whether there is an association between the distribution of ABO and Rh blood groups and the incidence of endometriosis in a cohort from Saudi Arabia and also to evaluate the potential risk factors related to endometriosis among the population.

Methods

This case-control study included women diagnosed with endometriosis (n = 44) who presented to King Abdulaziz University Hospital Obstetrics and Gynecology Clinic, Jeddah, Saudi Arabia, between 2010 and 2021. Women from the blood donors database of King Abdulaziz University Hospital were included as a control group (n = 184). The total sample size was 228. Demographic data, diagnosis method, ABO blood type, and Rh blood type were obtained from hospital records. In addition, data were collected from self-reported questionnaires, which included family history, dysmenorrhea, age of menarche, age of childbearing, number of children, history of abortion, parity, number of children, use of oral contraceptives for alleviating dysmenorrhea, iron deficiency, duration of menstrual flow, and volume of bleeding during menses. Odds ratio, Pearson chi-squared test (χ2), and independent t-test were used to analyze the associations between variables.

Results

Most of the participants had blood type O (n = 117, 51.3%), which was followed by blood type A (n = 59, 26.0%), and the majority were Rh+ (n = 215, 94.3%). There was no significant difference in the risk of endometriosis according to ABO (P = 0.237) and Rh (P = 0.283) blood types. However, endometriosis was found to have a significant relationship with dysmenorrhea, heavy bleeding during menses, history of abortion, long duration of menstrual flow, lower number of children, late pregnancy, and use of oral contraceptive pills to relieve dysmenorrhea (p ≤ 0.05).

Conclusions

The present results indicate that ABO and Rh blood types are not associated with the risk of endometriosis. However, there was a strong, significant association between endometriosis and other factors.

## Introduction

The ABO blood group system is based on the presence or absence of certain inherited antigenic glycoproteins on the surface of red blood cells [1]. In addition, based on the presence or absence of the D antigen, ABO blood groups are further classified as Rh positive or Rh negative [2]. These antigens play an important role in immunologic responses.

Endometriosis is an emerging chronic gynecological disease that has a profound impact on the quality of life. It is characterized by the development of tissue resembling the endometrium that is found at sites outside the uterine cavity; it causes pain and could even lead to infertility [3]. In addition, it is a chronic inflammatory condition that could lead to scarring, fibrosis, and adhesions in the affected area [4]. It affects 10% of reproductive-age women and girls worldwide (190 million) and is associated with infertility in 30% of affected individuals [5]. According to previous studies, early age at the first pregnancy, time lag between the first pregnancy and age at menarche, and infertility are risk factors for endometriosis. In addition, a significant association was found between higher levels of education, i.e., college or university degree, and the presence of endometriosis among subjects who underwent diagnostic laparoscopy and hysterectomy [6].

With regard to the influence of blood group on the development of endometriosis, a retrospective cohort study conducted at the Yale University Hospital among 230 women with endometriosis suggested that blood group A was associated with a 2.9-fold higher chance of developing endometriosis than other ABO blood groups [7]. Further, another study conducted in Paris in a French Caucasian population of 633 patients living in the same geographical area found that Rh-negative women were twice as likely to develop endometriosis [8]. In contrast to these findings, a study conducted at the Royan Institute and Arash Women’s Hospital in Iran among 433 women found that there was no significant link between blood groups (ABO/Rh) and the incidence of endometriosis; however, Iranian women with blood type O tended to have a lower chance of developing endometriosis than women with other blood types [9]. Further, another Iranian case-control study conducted among 539 participants revealed that there is no significant association between blood type and the occurrence of endometriosis [10]. Given these contradictory findings on the association between ABO and Rh blood groups and endometriosis incidence, further studies on more diverse and larger patient groups would be useful for clarifying whether blood type does influence the risk of endometriosis. Further, no studies have investigated the link between ABO and Rh blood types and endometriosis in Jeddah, Saudi Arabia. Therefore, to fill in this information gap, in this study, our primary Aim was to determine whether the distribution of ABO and Rh blood groups is associated with the incidence of endometriosis among women who presented to King Abdulaziz University Hospital (KAUH) Obstetrics and Gynecology Clinic, Jeddah, Saudi Arabia, and also evaluate the potential risk factors related to endometriosis among the population.

This article was previously posted to the Research Square preprint server on March 16, 2023.

## Materials and methods

Study design and setting

This was a case-control study carried out at KAUH, Jeddah, Saudi Arabia, between June 2021 and December 2021. KAUH is the largest tertiary care hospital in the Western region of Saudi Arabia. It has been accredited by many international accreditation bodies including the Accreditation Canada International, the Joint Commission International, the American Association of Blood Banks, and the College of American Pathologists [11]. The study was approved by the Biomedical Ethical Committee of KAUH (Ref No. 276-21). All study participants were notified about the research objectives, and their informed consent was obtained prior to commencement of the study.

Cases

Cases are defined as women with endometriosis who were diagnosed by laparoscopy, laparotomy, or by history taking and physical examination, from medical records from 2010 to 2020. A total of 144 cases were contacted to gain more information that was required for the study via a self-reported questionnaire. Consent was taken at the same time, and 44 consented to participate. Thus, the case group comprised 44 participants.

Controls

The control group was selected randomly from female blood donors in the KAUH blood bank over the past five years. The control group was matched with the endometriosis group by age ± 5 years and gender. We sent an electronic self-reported questionnaire, and informed consent was taken. Participants with incomplete records and who had chronic diseases were excluded. A total of 184 women were included in the control group.

Data collection 

Data were collected from patients’ hospital records from 2010 to 2020, as well as through a self-reported questionnaire. The research data collected from the hospital records included demographic characteristics, diagnosis method, blood group type (ABO), and Rh blood group (the ABO blood group and Rhesus factor were determined by standard techniques). We sent a self-reported questionnaire to participants for missing data that were needed for the research. The rest of the data were family history, dysmenorrhea, age of menarche, age of childbearing, number of children, history of abortion, need for oral contraceptive pills (OCPs) for alleviating dysmenorrhea, iron deficiency, duration of menstrual flow, and volume of bleeding during menses.

Recall and sampling bias

When information is gathered about people's past experiences or knowledge, there's always a chance of recall bias. This means that participants might not remember or report their experiences accurately. Also, the study might not have accurately represented the whole population if the participants had been more interested, educated, or accessible. It's possible that their level of interest and accessibility could have affected their willingness to participate in the study.

Statistical analysis

Microsoft Excel 2016 (Microsoft Corporation, Redmond, Washington, United States) was used for data entry, and IBM SPSS Statistics for Windows, Version 26.0 (Released 2019; IBM Corp., Armonk, New York, United States) was used to analyze the data. To assess the relationship between variables, qualitative data were expressed as numbers and percentages, and the odds ratio, independent samples t-test for continuous variables, and Pearson chi-squared test (χ2) for categorical variables were used to determine the significance of the results. Multivariate logistic regression analysis was also performed to assess the risk factors associated with menstrual irregularities. A p-value of 0.05 was considered to indicate statistical significance.

## Results

**Figure 1:**
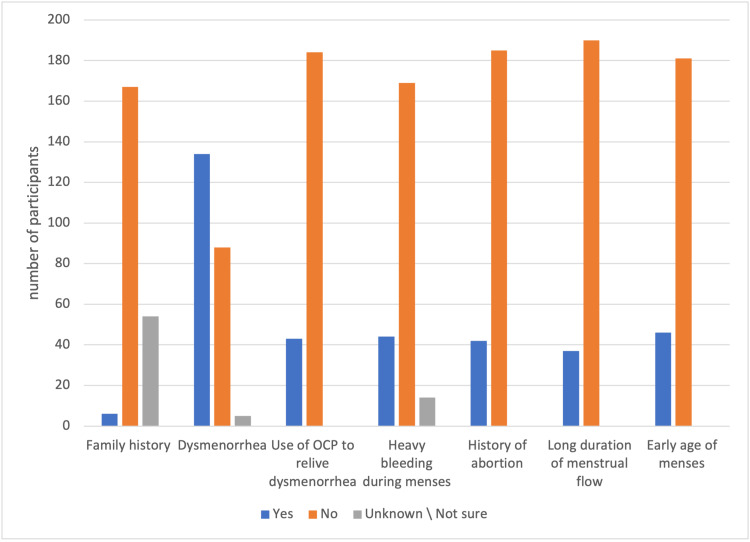
Family history of endometriosis and menstrual characteristics of the cohort (N = 228) OCP: oral contraceptive pill

The mean age of the participants included in the study was 30 ± 0.23 years, and the majority of the participants were Saudi nationals (n = 183, 80.3%). Most of the participants had blood type O (n = 117, 51.3%), which was followed by A (n = 59, 26.0%), and the majority were Rh+ (n = 215, 94.3%), as shown in Table 1. With regard to menstrual characteristics, the highest prevalence of endometriosis was recorded for participants who had dysmenorrhea (n = 134, 59%) and those who had never been pregnant (n = 149, 65.6%), as shown in Figure 1.

**Table 1:** . Distribution of demographic variables, blood groups, and obstetric characteristics in the study cohort (N = 228)

Parameters	Prevalence n (%)
Nationality	
Saudi	183 (80.3)
Non-Saudi	45 (19.8)
Blood group	
O	117 (51.3)
A	59 (26.0)
AB	12 (5.3)
B	40 (17.6)
RH factor	
Positive	215 (94.3)
Negative	13 (5.7)
Age of childbearing	
<20 years	23 (10.1)
20–30 years	48 (21.1)
31–40 years	8 (3.5)
Nulligravida (never been pregnant)	149 (65.4)
Number of children	
Nulliparous (never had children)	157 (68.9)
1–3	41 (18.0)
>3	30 (13.2)

**Figure 2:**
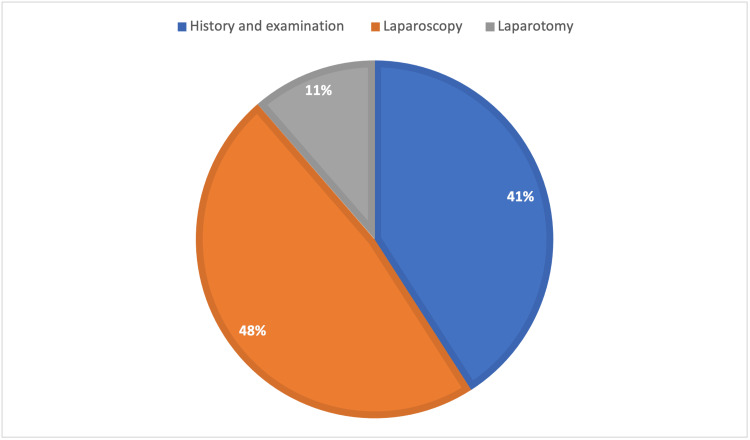
Proportion of methods used for diagnosis of endometriosis

The endometriosis group was divided according to the diagnosis method: history taking and physical examination (40.91%), laparoscopy (47.7%), and laparotomy (11.4%) (Figure 2). Thus, laparoscopy was the most common diagnostic method, and it was followed by history taking and physical examination.

The mean age of the endometriosis group was 35.30 ± 9.37 years, and the mean age of the control group was 29.1 ± 8.14 years (P > 0.001). Thus, women with endometriosis were significantly older than those without endometriosis. The most common ABO blood group was O, with a frequency of 51.1%. There was no significant association between ABO blood group and the presence of endometriosis (P = 0.227). The Rh+ blood group was present in 215 women (94.3%). There was no significant correlation between Rh+ blood group and the risk of endometriosis (P = 0.283) (Table 2).

**Table 2:** Distribution of combined covariate ABO and Rh blood groups in women with and without endometriosis ^a^p-value according to Pearson chi-square test; ^b^p-value according to multiple logistic regression analysis

Variable	Endometriosis group (n = 44), n (%)	Control group (n =184), n (%)	P-value^a^	OR	CI	P-value^b^
Blood group						
A	12 (27.3)	47 (25.7)	0.227	0.81	0.364–1.79	0.60
B	7 (15.9)	33 (18)		0.97	0.38–2.51	0.95
AB	5 (11.4)	7 (3.8)		0.29	0.08–1.00	0.05
O	20 (45.5)	97 (51.3)		Reference		
Rh factor						
Rh+	40 (90.9)	175 (95.1)	0.283	1.94	0.57–6.63	0.29
Rh-	4 (9.1)	9 (4.9)		Reference		

Menstrual characteristics and reproductive history in the case and control groups are shown in Table 3. A significant relationship was found between endometriosis and dysmenorrhea, heavy bleeding during menses, history of abortion, long duration of menstrual flow (more than seven days), lower number of children, late pregnancy, and use of OCPs to relieve dysmenorrhea (p ≤ 0.05). On the other hand, a non-significant relationship was found between the early age of menses (<11 years), iron deficiency anemia, and family history (p ≥ 0.05).

**Table 3:** Menstrual characteristics, reproductive history, and family history of the study cohort (N = 228) ^a^p-value according to Pearson chi-square; ^b^p-value according to multiple logistic regression analysis

Variable	Endometriosis group (n = 44), n (%)	Control group (n =184), n (%)	p-value^a^	OR	CI, p-value^b^
Dysmenorrhea					
Yes	32 (72.7)	102 (55.4)	0.015	0.50	0.24–1.03, 0.059
Heavy bleeding during menses					
Yes	22 (50.0)	22 (12.0)	0.000	0.17	0.03–0.83, 0.029
History of abortion					
Yes	13 (29.5)	29 (16.3)	0.072	0.465	0.22–0.99,0.047
Long duration of menstrual flow (>7 days)					
Yes	13 (29.5)	24 (13.0)	0.030	0.358	0.16–0.78,0.009
Early age of menses (<11 years)					
Yes	6 (13.6)	40 (21.7)	0.179	1.76	0.69–4.46, 0.234
Age of childbearing					
Before 20 years	7 (15.9)	15 (8.2)	0.000	0.28	0.1–0.77, 0.014
20–30 years	16 (36.4)	32 (17.5)		0.24	0.11–0.53, 0.000
31–40 years	5 (11.4)	3 (1.6)		0.07	0.02–0.33, 0.001
Nulligravida	16 (36.0)	133 (72.3)		Reference	
Number of children					
Nulliparous	19 (43.2)	138 (75.4)	0.004	Reference	
1–3	14 (31.8)	27 (14.7)		0.27	0.12–0.59, 0.001
>4	11 (25.0)	19 (10.3)		0.24	0.1–0.58, 0.001
Use of oral contraceptive pills to relive dysmenorrhea					
Yes	21 (47.7)	22 (12)	0.00	0.149	0.07–0.31, 0,000
Iron deficiency anemia					
Yes	14 (31.8)	33 (18.0)	0.099	0.48	0.22–1.01, 0.13
Family history					
Yes	3 (6.8)	3 (1.6)	0.642	0.85	0.39–1.84, 0.68

## Discussion

The aim of our study was to determine if the distribution of the ABO and Rh blood groups is associated with endometriosis in women who visited the KAUH Obstetrics and Gynecology Clinic in Jeddah, Saudi Arabia. This is the first study to examine this association in a cohort from Saudi Arabia. In a sample of 228 women, we investigated the association of ABO and Rh blood groups with the occurrence of endometriosis and several risk factors and symptoms associated with the disease. 

The gold standard for the diagnosis of endometriosis is visual inspection via laparoscopy or laparotomy, which was used in more than half of the participants in the current study. However, it is not always feasible in Saudi Arabia to use laparoscopy or laparotomy for routine medical care, and this procedure is not preferred by patients either. Therefore, the rest of the patients were diagnosed based on the clinical presentation, which is applied when the diagnosis can only be established by the patient's medical history, clinical examination, and ultrasound. According to the literature, board-certified gynecologists can identify endometriosis without laparoscopy in 80% of patients [12].

In the present study, we found that O was the most common ABO blood group, and the least frequent blood group was AB. Further, the most common Rh blood type was Rh+. These findings are in agreement with studies in Iran conducted by Malekzadeh et al. [9], Pourfathollah et al. [13], and Hosseini et al. [14]. With regard to the relationship between blood group and endometriosis risk, neither ABO nor Rh blood types were found to affect the risk of endometriosis, as reported in the study by Daliri et al. [10]. However, a study conducted by Borghese et al. in France reported that Rh- women are twice as likely to have endometriosis, while Demir et al. [15] reported that the risk of endometriosis in Rh+ women was significantly higher than that of the control group. The differences between these studies might be attributable to genetic variations among the study participants, as ethnicity, time, and geographic region are some of the known factors that affect the distribution of the Rh and ABO blood groups [9].

The findings of the current study demonstrated that the majority of the participants reported having dysmenorrhea, and a significant association was found between dysmenorrhea and the occurrence of endometriosis. Accordingly, it is known that endometriosis is the most common cause of secondary dysmenorrhea. In addition, we found that the incidence of heavy bleeding was higher among women with endometriosis. However, in contrast to our findings, other studies have demonstrated that there is no association between menstruation-related factors and endometriosis [6]. 

Studies have demonstrated that patients with a family history of endometriosis have a seven-fold higher risk of endometriosis [16]. In contrast, our results show that there was no significant association with family history. This is most likely the result of a lack of diagnosis or ignorance of the condition previously in the family. Our study also revealed a substantial link between endometriosis and the use of OCPs, which are used as the initial treatment for dysmenorrhea in young women [12]. In addition, late pregnancy issues and nulliparity were more prevalent in endometriosis-affected women; this has been seen in previous studies too [16]. Thus, endometriosis may be associated with the use of OCPs, late pregnancy, and nulliparity.

Limitations

Some limitations in our study should be considered. The study was limited to participants from one center and had a limited sample size, which may not represent the general population of women in Jeddah. Further, as part of the data were self-reported, some of the results may be skewed due to recall bias. Finally, as this is a case-control study, the causative relationship between factors could not be established, even though endometriosis showed a significant correlation with a few menstruation-related factors.

## Conclusions

This study found no significant association between the main blood groups (ABO and Rh) and the risk of endometriosis. However, the evidence is not sufficient to confirm/reject the hypothesis that the ABO system could be a genetic risk factor for the development of endometriosis, and further studies on larger samples and with more genetic factors are required.
